# Five microRNAs in plasma as novel biomarkers for screening of early-stage non-small cell lung cancer

**DOI:** 10.1186/s12931-014-0149-3

**Published:** 2014-11-25

**Authors:** Qing Geng, Tao Fan, Boyou Zhang, Wei Wang, Yao Xu, Hao Hu

**Affiliations:** Department of Thoracic Surgery, Renmin Hospital of Wuhan University, 238 Jie Fang Rd, Wuhan, 430060 China

**Keywords:** MicroRNAs, Plasma, Non-small cell lung cancer, Screening

## Abstract

**Background:**

In order to find novel noninvasive biomarkers with high accuracy for the screening of early-stage non-small cell lung cancer (NSCLC), we investigate the predictive power of 5 microRNAs (miR-20a, miR-145, miR-21, miR223 and miR-221) as potential biomarkers in early-stage NSCLC.

**Methods:**

In training set, 25 early-stage NSCLC patients and 25 matched healthy controls are included to assess the miRNA expression profile between early-stage NSCLC patients and healthy controls by real-time RT-PCR. We found that five of these miRNAs (miR-20a, miR-223, miR-21, miR-221 and miR-145) levels in NSCLC patients were significantly dysregulated compared with the healthy groups and thus were selected to validation set. Therefore, a validation experiment was further performed to investigate the potential predictive power of these five miRNAs based on 126 early-stage NSCLC patients, 42 NCPD patients and 60 healthy controls. The receiver operating characteristic (ROC) curves were generated for the five miRNAs.

**Results:**

ROC curve analyses suggested that these five plasma miRNAs could be promising biomarkers for NSCLC, with relatively high AUC values as follows: miR-20a, 0.89 with 95% CI of [0.85-0.93]; miR-223, 0.94 with 95% CI of [0.91-0.96]; miR-21, 0.77 with 95% CI of [0.71-0.83]; miR-155, 0.92 with 95% CI of [0.89-0.96]; miR-145, 0.77 with 95% CI of [0.71-0.83]. Stratified analyses indicated that plasma miR-20a, miR-223, miR-21 and miR-145 showed better predictive value in smokers than in non-smokers, while miR-155 might be more suitable for non-smokers. In addition, all of these five miRNAs could differentiate NSCLC from controls with a higher accuracy in advanced stage and squamous carcinoma subgroups.

**Conclusions:**

In conclusion, our study suggested that five plasma miRNAs (miR-20a, miR-145, miR-21, miR-223 and miR-221) can be used as promising biomarkers in early screening of NSCLC. Nevertheless, further validation and optimizing improvement should be performed on larger sample to confirm our results.

## Introduction

Lung cancer causes extremely high mortality of cancer death worldwide, almost 85% of which are from non-small cell lung cancer (NSCLC) [1,2]. It was estimated that NSCLC may remain to be one of leading cause of deaths in the next 50 years [3]. Early detection is the most effective way to relieve this threatening disease, since five-year survival rate is ~80% in early stages (stage I/II) but drops sharply to ~14% in advanced stages (stage III/IV) [1]. Unfortunately, still 75% of NSCLC cases are diagnosed in advanced stages due to the lack of effective early diagnostic methods [3].

Currently, pathological diagnosis based on biopsies remains to be the standard methods for early-stage NSCLC detection, such as bronchoscopy, which have an advantage over the other methods since it can dynamically monitor the aberrant conditions of lung, however, the invasive nature of this technique poses a potential risk on human body [4,5]. Imaging techniques, such as chest X-ray and computed tomography (CT), are also used to detect early-stage NSCLC [6-9], but the exposure to the radiation may do harm to health. Considering these limitations of the expensive methods mentioned above, scientists turn to seek noninvasive screening markers for early-stage NSCLC diagnosis. Several protein biomarkers have been found as noninvasive and cost-effective diagnostic tools for early-stage NSCLC, such as CA-125, CA19-9, CEA, CYFRA21-1, chromogranin A, NSE and TPS [10,11]. However, the limited sensitivity and specificity hampered their further application and development. Therefore, it is significantly urgent to develop novel noninvasive biomarkers with high accuracy for the screening of early-stage NSCLC.

Recently, a new group of RNA regulatory genes, microRNAs (miRNAs), has been discovered to be closely associated with various human cancers, including NSCLC [12]. MiRNAs regulate post-transcriptionally the expression of a wide range of genes, which play an important role in controlling cell proliferation, differentiation, and apoptosis [13-15]. Besides, accumulating studies have proven that miRNAs can serve as tumor suppressors due to its dysregulated expression in cancer development and progression by inhibiting the translation of 3′-untranslated region of messenger RNAs [15]. As tumor-related genes, miRNAs are considered to have the potential diagnostic value in the cancer detection. Furthermore, miRNAs as cancer biomarkers show additional advantages: (1) miRNAs are easy to extract since they universally exist in tissue or body fluids, such as serum, plasma, urine, etc.; (2) miRNAs show strong stability and resistance to boiling, extended storage, RNase degradation, extremes of PH, and multiple freeze-thaw cycles [16]. Based on the above evidences, it is obvious that miRNAs have great potential as noninvasive and easy-operating methods in the cancer detection.

Over the past few years, several studies have indicated that some miRNAs can serve as potential biomarkers for NSCLC with high accuracy, as its expression levels between NSCLC patients and healthy controls show significant differences [11,17-19]. However, there are few studies focusing on the potential predictive value of miRNAs for early-stage NSCLC. As we know, early detection is the most effective way to reduce the high mortality of NSCLC, and we carried out this study to investigate potential miRNAs for early-stage NSCLC diagnosis. In this study, we selected 12 candidate plasma miRNAs mentioned in these studies [11,17-21], which are claimed to have predictive value in early-stage NSCLC, including miR-30d, miR-383, miR-20a, miR-145, miR-221, miR-25, miR-223, miR-21, miR-126, miR-155, miR-182, and miR-210. We first investigated the expression levels of miRNAs in plasma for early-stage NSCLC using real-time quantitative reverse transcription PCR (real-time qRT-PCR). And then we picked out five miRNAs (miR-20a, miR-145, miR-21, miR223 and miR-221) which showed significant differences in the expression levels between cancer patients and controls to perform further investigation to confirm their diagnostic value. The application of these plasma miRNAs as biomarkers for early-stage NSCLC screening will be interpreted in this study.

## Methods and materials

### Selection of plasma miRNAs

According to a large number of relevant articles which have reported the diagnostic value of miRNAs for NSCLC [11,17-21], we chose 12 miRNAs as our research target, including miR-30d, miR-383, miR-20a, miR-145, miR-221, miR-25, miR-223, miR-21, miR-126, miR-155, miR-182, and miR-210. The inclusion criteria can be summarized as follows: (1) they have been proven to exist in plasma; (2) The expression levels of these miRNAs showed significant differences in between NSCLC patients and healthy controls; (3) they suggest potential diagnostic value in early stage NSCLC detection.

### Ethic statement and patient samples

In this double-blind experiment, all the subjects were selected from Renmin Hospital of Wuhan University (Wuhan, China) and we have obtained informed consents from all of the participants with the approval from the ethics committee of Renmin Hospital of Wuhan University. In total, 151 early stage NSCLC patients were recruited. Gold standard methods, including lung biopsy specimens and imaging techniques, were applied to confirm the histopathological features and tumor stages of NSCLC patients. None of the patients have ever received surgery or chemotherapy before. 85 healthy controls were selected including smokers and non-smokers but with no history of pulmonary diseases. 42 non-cancerous pulmonary disease (NCPD) patients were further recruited as a control group, including 25 chronic obstructive pulmonary disease (COPD) patients and 17 benign pulmonary nodule (BPN) patients. All the necessary information of patients and healthy controls is provided in Table 1.

**Table 1:** **Demographic and clinical characteristics of early-stage NSCLC patients and healthy controls**

	**Training set**			**Validation set**			
	**NSCLC (n = 25)**	**Healthy (n = 25)**	***P*** **-value**	**NSCLC (n = 126)**	**NCPD (n = 42)**	**Healthy (n = 60)**	***P*** **-value**
**Age**							
≤60	14	13	0.777	79	26	35	0.847
>60	11	12		47	16	25	
**Sex**							
Male	15	12	0.395	87	29	36	0.443
Female	10	13		39	13	24	
**Smoking status**							
Current	9	7	0.807	54	21	17	*0.004*
Former	13	14		35	13	11	
Never	3	4		37	8	32	
**TNM stage**							
Stage I	9	-	-	54	-	-	-
Stage II	16	-		72	-	-	
**Histological type**							
Adenocarcinoma	8	-	-	45	-	-	-
Squamous carcinoma	13	-		64	-	-	
Others	4	-		17	-	-	

NSCLC, non-small cell lung cancer; NCPD, non-cancerous pulmonary disease.

### Study design: training set and validation set

This study was divided into two sets, training set and validation set. In the training set, we chose 25 early-stage NSCLC patients and 25 age- and gender-matched healthy controls to compare the expression profile of these 12 miRNAs between NSCLC patients and healthy controls. There are no significant differences in age, gender and smoking status. According to the training set results, we then find some of the miRNAs that show statistically significant differences in expression levels, and performed a validation experiment to further investigate the diagnostic proficiency of these miRNAs. In the validation set, plasma samples were drawn from all participants complying with the World Health Organization (WHO) categories, including 126 early-stage NSCLC patients, 42 NCPD patients and 60 healthy controls.

### RNA isolation and qRT-PCR analysis for miRNAs

Blood samples (5 mL per subjects) were drawn and stored into BD Vacutainer spray-coated K2EDTA Tubes (BD, Franklin Lakes, NJ, USA) with EDTA inside. Then each blood sample was centrifuged at 2,000 × g for 10 min at 4°C aiming at isolating the plasma from blood, which was then immediately transferred into a new Eppendorf tube and frozen to −80°C until RNA extraction process. In all 50 μL RNA was isolated from each 500 μL plasma sample.

After solution, 20 μL reactions including miRNA-specific reverse primers and transcription (RT) mixture were added to plasma RNA to initiate the transcription of these 12 miRNAs. These procedures were performed on miScript SYBR Green PCR kit (Qiagen, Germany) with abidance by the manufacturer’s protocol. Quantitative PCR was carried out on a Bio-Red IQ5 Multi-color RT-PCR Detection System (Bio-Red, Hercules, CA, USA). Comparative cycle threshold (Ct) was calculated to define the expression level of these miRNAs. MiR-16 was selected as internal reference, since accumulating studies have reported that it is relatively stable in the test environment [22-25]. The expression level of each included miRNA can be determined by this equation: 2^-△△Ct^, ΔCt = Ct (reference miR-16) - Ct (miRNA). Each qRT-PCR test was conducted in triplicate. Besides, in order to rule out the effects from test environment, one no-template control and two interpolate controls were performed alone for each sample.

### Statistics analysis

The significances of 12 included miRNAs were appraised by Mann–Whitney test. In the training set, the expression levels of these 12 miRNAs in between patients and healthy controls were detected individually, with a *P* value less than 0.05 showing no significant diagnostic value in differentiating the early-stage NSCLC patients from healthy controls. After selecting those effective miRNAs, we further conducted experiment based on each miRNA. Except for the same tests mentioned above which should be performed, additional tests should be carried out to comprehensively evaluate the diagnostic value of these miRNAs as biomarkers for early-stage NSCLC detection. Thus, receiver operating characteristic (ROC) curve was established to interpret the ability of miRNA in discriminating patients from healthy controls. The area under the curve (AUC), sensitivity and specificity at the optimal cutoff were computed, which would validate the diagnostic application of these effective miRNAs as cancer biomarkers. All the *P* values were bilaterally shown, with a value less than 0.05 indicating statistically significance.

## Results

### Demographic and clinicopathological characteristics of subjects

Of the 25 NSCLC patients in the training set, 9 patients are at stage I and 16 at stage II; 8 are suffered from adenocarcinoma, 13 with squamous carcinoma and 4 with other subtype NSCLC. A total of 25 healthy controls were selected for training set. The age, sex and smoking habit of healthy controls were well matched with NSCLC patients. In the validation set, there were in total 126 NSLCL patients, 42 NCPD patients and 60 healthy controls. Although the case and control groups were well matched for age (*P* = 0.847) and sex (*P* = 0.443) as shown in revised Table 1, smoking habit (*P* = 0.004) as an uncontrollable variable was not matched well between NSCLC patients and controls. Therefore, we conducted further stratified analysis according to smoking habit, which classified both NSCLC patients and controls into two groups, such as smokers (former and current smokers) and non-smokers. These demographic and clinicopathological characteristics for subjects were listed in Table 1 in details.

### Evaluation of 12 candidate miRNAs as biomarkers for NSCLC screening in training set

In the training set, we tested 12 candidate miRNAs in plasma samples by qRT-PCR in both cases and controls. The relative expression of 12 miRNAs was measured in plasma RNA for 25 NSCLC patients and 25 healthy controls, as shown in Table 2. We found that all miRNAs exhibited an up-regulated trend in NSCLC patients, but only five miRNAs (miR-20a, miR-223, miR-21, miR-221 and miR-145) showed significant differences between NSCLC patients and healthy controls. Hence, these five miRNAs were further investigated in a large-scale sample in validation set in order to validate their diagnostic accuracy.

**Table 2:** **Expression levels of 12 plasma miRNAs between early-stage NSCLC patients and healthy controls**

**miRNAs**	**Expression**	**NSCLC/Healthy**	***P*** **value**
miR-20a	↑	12.34 ± 0.78	*1.45 × 10* ^*−3*^
miR-223	↑	5.67 ± 0.56	*2.34 × 10* ^*−3*^
miR-21	↑	5.09 ± 0.53	*3.31 × 10* ^*−5*^
miR-155	↑	4.34 ± 0.3	*1.67 × 10* ^*−3*^
miR-221	↑	1.32 ± 0.29	0.063
miR-25	↑	1.26 ± 0.28	0.082
miR-182	↑	1.03 ± 0.27	0.723
miR-30d	↑	0.98 ± 0.25	0.459
miR-126	↑	0.87 ± 0.24	0.239
miR-320	↑	1.84 ± 0.77	0.071
miR-210	↑	0.57 ± 0.15	0.563
miR-145	**↑**	20.24 ± 0.83	*3.56 × 10* ^*−4*^

### Evaluation of 5 novel miRNAs as biomarkers for NSCLC screening in validation set

**Figure 1:**
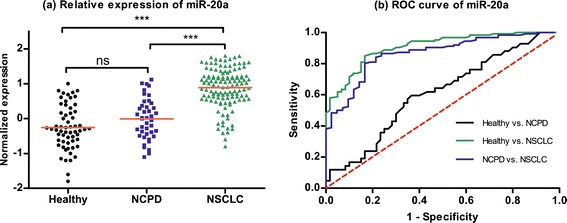
**Scatter plot of expression levels (a) and Receiver operator characteristic (ROC) curve (b) analysis of plasma miR-20a.**

**Figure 2:**
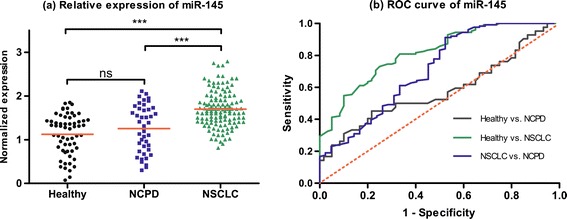
**Scatter plot of expression levels (a) and Receiver operator characteristic (ROC) curve (b) analysis of plasma miR-223.**

**Figure 3:**
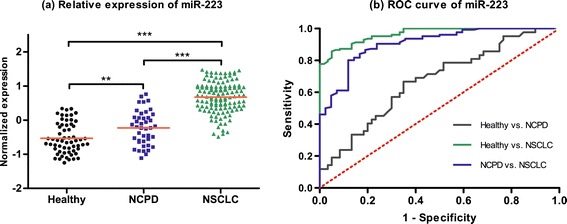
**Scatter plot of expression levels (a) and Receiver operator characteristic (ROC) curve (b) analysis of plasma miR-21.**

**Figure 4:**
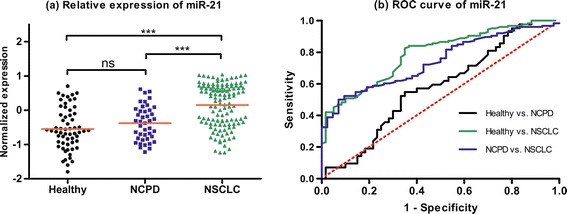
**Scatter plot of expression levels (a) and Receiver operator characteristic (ROC) curve (b) analysis of plasma miR-155.**

**Figure 5:**
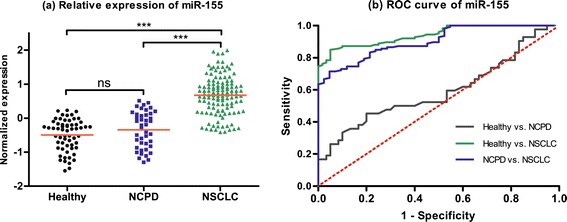
**Scatter plot of expression levels (a) and Receiver operator characteristic (ROC) curve (b) analysis of plasma miR-145.**

In validation set, we choose these five miRNAs (miR-20a, miR-223, miR-21, miR-221 and miR-145) as novel biomarkers for NSCLC screening based on 126 NSCLC patients, 42 NCPD patients and 60 healthy controls. We first pondered the predictive application of these five miRNAs by comparing the relative expression in plasma between NSCLC patients and two control groups. As shown in Figures 1, 2, 3, 4 and 5, there were different expression of these five miRNAs between NSCLC patients and healthy controls (all *P* < 0.001), as well as NSCLC patients and NCPD controls. However, no significant difference was observed between NCPD patients and healthy controls for miR-20a, miR-21, miR-221 and miR-145 (all *P* > 0.05), except for miR-223 (*P* < 0.01).

ROC curve analyses were conducted to figure out the sensitivity and specificity of these five miRNAs as biomarkers in the screening of NSCLC (Table 3). For the training set, the AUC of each ROC curve of these 5 miRNAs ranged from 0.79 to 0.96, as follows: miR-20a, 0.91 with 95% confidence interval (CI) of [0.86-0.95]; miR-223, 0.96 with 95% CI of [0.94-0.98]; miR-21, 0.79 with 95% CI of [0.73-0.86]; miR-155, 0.94 with 95% CI of [0.91-0.97]; miR-145, 0.82 with 95% CI of [0.75-0.88]. For the validation set, similar results were obtained, as follows: miR-20a, 0.89 with 95% CI of [0.85-0.93]; miR-223, 0.94 with 95% CI of [0.91-0.96]; miR-21, 0.77 with 95% CI of [0.71-0.83]; miR-155, 0.92 with 95% CI of [0.89-0.96]; miR-145, 0.77 with 95% CI of [0.71-0.83].

**Table 3:** **Sensitivity and specificity and AUC of 5 identified miRNAs in detecting early-stage NSCLC**

**MiRNA**	**Training set (NSCLC vs. Healthy)**			**Validation set (NSCLC vs. Control)**		
	**Sensitivity (95% CI)**	**Specificity (95% CI)**	**AUC (95% CI)**	**Sensitivity (95% CI)**	**Specificity (95% CI)**	**AUC (95% CI)**
miR-20a	0.84 [0.76-0.90]	0.83 [0.71-0.92]	0.91 [0.86-0.95]	0.83 [0.74-0.89]	0.81 [0.72-0.88]	0.89 [0.85-0.93]
miR-223	0.87 [0.80-0.93]	0.86 [0.75-0.94]	0.96 [0.94-0.98]	0.87 [0.80-0.92]	0.86 [0.78-0.92]	0.94 [0.91-0.96]
miR-21	0.70 [0.61-0.78]	0.68 [0.55-0.80]	0.79 [0.73-0.86]	0.67 [0.54-0.72]	0.68 [0.59-0.77]	0.77 [0.71-0.83]
miR-155	0.87 [0.80-0.93]	0.87 [0.75-0.94]	0.94 [0.91-0.97]	0.86 [0.78-0.91]	0.84 [0.76-0.91]	0.92 [0.89-0.96]
miR-145	0.73 [0.64-0.81]	0.75 [0.62-0.85]	0.82 [0.75-0.88]	0.70 [0.61-0.78]	0.68 [0.58-0.76]	0.77 [0.71-0.83]

Furthermore, we conducted stratified analyses in different clinical-pathological subgroups, according to smoking habit (smoker vs. non-smoker), tumor stage (stage I-II vs. stage III-IV), and histological type (adenocarcinoma vs. squamous carcinoma), as shown in Table 4. Our results indicated that miR-20a, miR-223, miR-21 and miR-145 showed better diagnostic performance in smokers than in non-smokers, while miR-155 might be more suitable for non-smokers. In addition, all of these five miRNAs could differentiate NSCLC from controls with a higher accuracy in advanced stage and squamous carcinoma subgroups.

**Table 4:** **Subgroup analysis of predictive value for the 5 identified miRNAs in validation set**

**MiRNAs**	**NSCLC vs. Healthy**			**NSCLC vs. NCPD**			**NCPD vs. Healthy**		
	**SEN**	**SPE**	**AUC**	**SEN**	**SPE**	**AUC**	**SEN**	**SPE**	**AUC**
**miR-20a**	0.81	0.85	0.91	0.81	0.81	0.86	0.60	0.63	0.61
Smokers	0.89	0.89	0.98	0.85	0.85	0.94	0.64	0.52	0.61
Non-smokers	0.63	0.63	0.64	0.70	0.73	0.75	0.66	0.67	0.68
Stage I	0.77	0.76	0.84	0.76	0.76	0.77	0.63	0.59	0.61
Stage II	0.98	0.98	0.99	0.98	0.97	0.99	0.60	0.59	0.61
AC	0.87	0.83	0.90	0.84	0.81	0.85	0.61	0.59	0.62
SC	0.97	0.97	0.98	0.92	0.90	0.98	0.63	0.59	0.60
**miR-223**									
Smokers	0.85	0.85	0.94	0.93	0.90	0.98	0.65	0.65	0.69
Non-smokers	0.78	0.78	0.93	0.78	0.75	0.89	0.72	0.75	0.73
Stage I	0.72	0.70	0.86	0.65	0.64	0.70	0.65	0.64	0.67
Stage II	0.97	0.97	0.98	0.94	0.95	0.98	0.65	0.64	0.67
AC	0.80	0.82	0.91	0.73	0.71	0.77	0.65	0.62	0.67
SC	0.95	0.93	0.98	0.92	0.93	0.98	0.65	0.64	0.67
**miR-21**									
Smokers	0.83	0.81	0.87	0.78	0.75	0.84	0.63	0.63	0.64
Non-smokers	0.55	0.50	0.57	0.53	0.52	0.55	0.57	0.58	0.59
Stage I	0.67	0.67	0.69	0.57	0.57	0.61	0.56	0.57	0.58
Stage II	0.87	0.88	0.96	0.88	0.88	0.95	0.57	0.56	0.58
AC	0.60	0.60	0.63	0.51	0.50	0.56	0.57	0.57	0.58
SC	0.91	0.90	0.97	0.92	0.90	0.97	0.56	0.57	0.58
**miR-155**									
Smokers	0.86	0.82	0.88	0.78	0.76	0.88	0.52	0.51	0.53
Non-smokers	0.91	0.93	0.98	0.89	0.88	0.98	0.65	0.63	0.64
Stage I	0.80	0.80	0.91	0.76	0.76	0.84	0.55	0.52	0.59
Stage II	0.94	0.95	0.97	0.83	0.83	0.95	0.55	0.52	0.60
AC	0.87	0.87	0.93	0.80	0.78	0.86	0.55	0.52	0.59
SC	0.92	0.90	0.96	0.81	0.81	0.93	0.52	0.52	0.59
**miR-145**									
Smokers	0.96	0.96	0.98	0.95	0.90	0.97	0.57	0.58	0.68
Non-smokers	0.81	0.84	0.90	0.63	0.62	0.79	0.60	0.62	0.63
Stage I	0.59	0.57	0.62	0.51	0.50	0.55	0.52	0.52	0.58
Stage II	0.91	0.90	0.96	0.76	0.73	0.87	0.52	0.52	0.58
AC	0.71	0.70	0.77	0.57	0.55	0.59	0.55	0.50	0.58
SC	0.95	0.95	0.97	0.79	0.79	0.89	0.52	0.52	0.58

AC, adenocarcinoma; SC, squamous carcinoma.

## Discussion

Although NSCLC has high mortality rate among cancers, the NSCLC patients are more likely to survive if they are diagnosed and received treatment at its early stage, since the five-year survival rate of early-stage cancer can reach up to 80%. Current diagnostic methods for NSCLC mainly consist of pathological biopsy, imaging diagnosis and protein biomarkers, which however, suffer from a lot of limitations. Fortunately, the discovery of miRNAs would open a door to the promising perspective of accurate early diagnosis of NSCLC as they are noninvasive tumor-specific biomarkers to discriminate patients from healthy controls with high sensitivity and specificity.

Over the past few years, scientists have found sufficient evidences to confirm the aberrant expression of miRNAs between cancer patients and healthy people which have a strong correlation with cancer development [26,27]. Chen et al. found a significant difference in the expression levels of serum miRNAs between NSCLC patients and healthy controls [11]. Tang et al. revealed that miRs-21, 145 and −155 can serve as noninvasive screening tool in the early detection of lung cancer with relatively high accuracy [21]. Subsequently, increasing studies have investigated the diagnostic value of miRNAs for early-stage lung cancer. Compared with these previous studies, our present study has several advantages. Firstly, we concentrated on the detection of early-stage NSCLC by using miRNAs as biomarkers, which was a novel research field aiming at reducing the high mortality through early detection. Secondly, 12 miRNAs were included in our research and 5 miRNAs were confirmed to have diagnostic value, thus expanding the investigation number of miRNAs, which would make the interpretation more comprehensively and systematically.

Our results suggested that NSCLC patients and the healthy people have the aberrant expression levels of these five plasma miRNAs (miR-20a, miR-223, miR-21, miR-221 and miR-145). The data from our study demonstrated that the each single miRNA present high sensitivity and specificity in the detection process. Despite of different expression levels, all of these five miRNAs were validated to have potential to discriminate the early-stage NSCLC patients from healthy controls. However, qRT-PCR analyses suggested that there was no significant expression difference between NCPD patients and healthy controls for all five miRNA. During the research, we found that the functional pattern and expression level of these miRNAs can lead to the pathological alterations, due to their underlying functions tumor suppressors or oncogenes, which makes them effective indicators in cancer diagnosis [28-31].

Among these five miRNAs, miR-20a, −223 and miR-155 were demonstrated as the most sensitive and specific biomarkers in the detection of NSCLC, showing the superior accuracy. Previous findings have confirmed that miR-20a can inhibit *E2F1* directly, which is a transcription factor associated with the lung cancer cell growth [32], thus making miR-20a helpful for the early diagnosis of NSCLC. As for miR-155, scientists have proven that restoration of miR-155 represses the growth of NSCLC malignant cells in the epidermal growth factor receptor mutant, which can serve as reasonable explanation for miR-155 as a NSCLC biomarker at present [33,34].

In our study, we have demonstrated these five miRNAs in plasma showed dysregulated expression in NSCLC, suggesting they can serve as biomarkers in precise clinical diagnosis of early-stage NSCLC. However, some limitations in our tests needed to be noticed. Firstly, in order to acquire a better and deeper understanding of the diagnostic performance of these five miRNAs in the early detection of NSCLC, more complementary researches on the regulatory mechanism of miRNA in cancer should be needed to confirm our results. Secondly, the combination of these miRNAs should be performed to find out the most effective biomarker group and improve the diagnostic accuracy, since the combination of miRNAs as screening tools may be more sensitive and specific than single miRNA [18,21]. Besides, a larger sample size will be required to produce a more convincing result in future. Ignoring these limitations, our study still demonstrated authorized clinical diagnostic methods for early-stage NSCLC.

## Conclusions

To sum up, sufficient evidences have been provided in our study to show the aberrant expression in the levels of plasma miR-20a, miR-223, miR-21, miR-221 and miR-145 in early-stage NSCLC patients. The clinical diagnostic value of each miRNA has been further evaluated, which indicated that these five miRNAs can serve as noninvasive biomarkers in the detection of early-stage NSCLC individually. Nevertheless, further validation study should be performed on larger sample to confirm our results.

## Abbreviations

NSCLC: Non-small cell lung cancer
miRNAs: microRNAs
ROC: Receiver operating characteristic
AUC: Area under the curve
real-time qRT-PCR: Real-time quantitative reverse transcription PCR

## References

[1] Jemal A, Bray F, Center MM, Ferlay J, Ward E, Forman D (2011). Global cancer statistics. CA Cancer J Clin.

[2] Ettinger DS, Akerley W, Bepler G, Blum MG, Chang A, Cheney RT, Chirieac LR, D’Amico TA, Demmy TL, Ganti AK, Govindan R, Grannis FW, Jahan T, Jahanzeb M, Johnson DH, Kessinger A, Komaki R, Kong FM, Kris MG, Krug LM, Le QT, Lennes IT, Martins R, O’Malley J, Osarogiagbon RU, Otterson GA, Patel JD, Pisters KM, Reckamp K, Riely GJ (2010). Non-small cell lung cancer. J Natl Compr Canc Netw.

[3] Foss KM, Sima C, Ugolini D, Neri M, Allen KE, Weiss GJ (2011). miR-1254 and miR-574-5p: serum-based microRNA biomarkers for early-stage non-small cell lung cancer. J Thorac Oncol.

[4] Hirsch FR, Franklin WA, Gazdar AF, Bunn PA (2001). Early detection of lung cancer: clinical perspectives of recent advances in biology and radiology. Clin Cancer Res.

[5] Ikeda N, Hayashi A, Iwasaki K, Honda H, Tsuboi M, Usuda J, Kato H (2007). Comprehensive diagnostic bronchoscopy of central type early stage lung cancer. Lung Cancer.

[6] Swensen SJ, Jett JR, Hartman TE, Midthun DE, Mandrekar SJ, Hillman SL, Sykes AM, Aughenbaugh GL, Bungum AO, Allen KL (2005). CT screening for lung cancer: five-year prospective experience. Radiology.

[7] Investigators NYELCAP (2007). CT screening for lung cancer: diagnoses resulting from the New York early lung cancer action project. Radiology.

[8] Aberle DR, Adams AM, Berg CD, Black WC, Clapp JD, Fagerstrom RM, Gareen IF, Gatsonis C, Marcus PM, Sicks J (2011). Reduced lung-cancer mortality with low-dose computed tomographic screening. N Engl J Med.

[9] de González AB, Kim KP, Berg CD (2008). Low-dose lung computed tomography screening before age 55: estimates of the mortality reduction required to outweigh the radiation-induced cancer risk. J Med Screen.

[10] Tarro G, Perna A, Esposito C (2005). Early diagnosis of lung cancer by detection of tumor liberated protein. J Cell Physiol.

[11] Chen X, Hu Z, Wang W, Ba Y, Ma L, Zhang C, Wang C, Ren Z, Zhao Y, Wu S (2012). Identification of ten serum microRNAs from a genome‐wide serum microRNA expression profile as novel noninvasive biomarkers for nonsmall cell lung cancer diagnosis. Int J Cancer.

[12] Bartel DP (2004). MicroRNAs: genomics, biogenesis, mechanism, and function. Cell.

[13] Hwang H, Mendell J (2006). MicroRNAs in cell proliferation, cell death, and tumorigenesis. Br J Cancer.

[14] Kumar MS, Lu J, Mercer KL, Golub TR, Jacks T (2007). Impaired microRNA processing enhances cellular transformation and tumorigenesis. Nat Genet.

[15] Sassen S, Miska EA, Caldas C (2008). MicroRNA—implications for cancer. Virchows Arch.

[16] Brase JC, Wuttig D, Kuner R, Sultmann H (2010). Serum microRNAs as non-invasive biomarkers for cancer. Mol Cancer.

[17] Chen X, Ba Y, Ma L, Cai X, Yin Y, Wang K, Guo J, Zhang Y, Chen J, Guo X (2008). Characterization of microRNAs in serum: a novel class of biomarkers for diagnosis of cancer and other diseases. Cell Res.

[18] Shen J, Todd NW, Zhang H, Yu L, Lingxiao X, Mei Y, Guarnera M, Liao J, Chou A, Lu CL (2010). Plasma microRNAs as potential biomarkers for non-small-cell lung cancer. Lab Invest.

[19] Boeri M, Verri C, Conte D, Roz L, Modena P, Facchinetti F, Calabrò E, Croce CM, Pastorino U, Sozzi G (2011). MicroRNA signatures in tissues and plasma predict development and prognosis of computed tomography detected lung cancer. Proc Natl Acad Sci.

[20] Yang XQ, Zhang YH, Sun P, Deng P, Fan J, Shen JH, Zhu GY, Gu MF (2013). [Diagnostic value of the detection of microRNAs in sputum of patients with non-small cell lung caner]. J Clin Pulmon Med.

[21] Tang D, Shen Y, Wang M, Yang R, Wang Z, Sui A, Jiao W, Wang Y (2013). Identification of plasma microRNAs as novel noninvasive biomarkers for early detection of lung cancer. Eur J Cancer Prev.

[22] Wong T-S, Liu X-B, Wong BY-H, Ng RW-M, Yuen AP-W, Wei WI (2008). Mature miR-184 as potential oncogenic microRNA of squamous cell carcinoma of tongue. Clin Cancer Res.

[23] Lawrie CH, Gal S, Dunlop HM, Pushkaran B, Liggins AP, Pulford K, Banham AH, Pezzella F, Boultwood J, Wainscoat JS (2008). Detection of elevated levels of tumour‐associated microRNAs in serum of patients with diffuse large B‐cell lymphoma. Br J Haematol.

[24] Liu CJ, Kao SY, Tu HF, Tsai MM, Chang KW, Lin SC (2010). Increase of microRNA miR‐31 level in plasma could be a potential marker of oral cancer. Oral Dis.

[25] Kroh EM, Parkin RK, Mitchell PS, Tewari M (2010). Analysis of circulating microRNA biomarkers in plasma and serum using quantitative reverse transcription-PCR (qRT-PCR). Methods.

[26] Bi CL, Chng WJ (2011). miRNA deregulation in multiple myeloma. Chin Med J (Engl).

[27] Wu DG, Wang YY, Fan LG, Luo H, Han B, Sun LH, Wang XF, Zhang JX, Cao L, Wang XR, You YP, Liu N (2011). MicroRNA-7 regulates glioblastoma cell invasion via targeting focal adhesion kinase expression. Chin Med J (Engl).

[28] Lu J, Getz G, Miska EA, Alvarez-Saavedra E, Lamb J, Peck D, Sweet-Cordero A, Ebert BL, Mak RH, Ferrando AA, Downing JR, Jacks T, Horvitz HR, Golub TR (2005). MicroRNA expression profiles classify human cancers. Nature.

[29] Shenouda SK, Alahari SK (2009). MicroRNA function in cancer: oncogene or a tumor suppressor?. Cancer Metastasis Rev.

[30] Garofalo M, Condorelli G, Croce C, Condorelli G (2009). MicroRNAs as regulators of death receptors signaling. Cell Death Diff.

[31] Kouhkan F, Alizadeh S, Kaviani S, Soleimani M, Pourfathollah AA, Amirizadeh N, Abroun S, Noruzinia M, Mohamadi S (2011). miR-155 down regulation by LNA inhibitor can reduce cell growth and proliferation in PC12 cell line. Avicenna J Med Biotechnol.

[32] O’Donnell KA, Wentzel EA, Zeller KI, Dang CV, Mendell JT (2005). c-Myc-regulated microRNAs modulate E2F1 expression. Nature.

[33] Lynch TJ, Bell DW, Sordella R, Gurubhagavatula S, Okimoto RA, Brannigan BW, Harris PL, Haserlat SM, Supko JG, Haluska FG, Louis DN, Christiani DC, Settleman J, Haber DA (2004). Activating mutations in the epidermal growth factor receptor underlying responsiveness of non-small-cell lung cancer to gefitinib. N Engl J Med.

[34] Yanaihara N, Caplen N, Bowman E, Seike M, Kumamoto K, Yi M, Stephens RM, Okamoto A, Yokota J, Tanaka T, Calin GA, Liu CG, Croce CM, Harris CC (2006). Unique microRNA molecular profiles in lung cancer diagnosis and prognosis. Cancer Cell.

